# Buruli ulcer in Nigeria: results of a pilot case study in three rural districts

**DOI:** 10.1186/s40249-016-0119-8

**Published:** 2016-04-22

**Authors:** Kingsley N. Ukwaja, Anthony O. Meka, Alphonsus Chukwuka, Kingsley B. Asiedu, Kristina L. Huber, Miriam Eddyani, Joseph N. Chukwu, Moses C. Anyim, Charles C. Nwafor, Daniel C. Oshi, Nelson O. Madichie, Ngozi Ekeke, Martin Njoku, Kentigern Ntana

**Affiliations:** Department of Medicine, Federal Teaching Hospital , FMC Rd, Abakaliki, Ebonyi State Nigeria; Medical Department, German Leprosy and TB Relief Association, Enugu, Enugu State Nigeria; St Benedict’s Tuberculosis and Leprosy Rehabilitation Hospital, Ogoja, Cross River State Nigeria; Global Buruli Ulcer Initiative, Department of Control of Neglected Tropical Diseases, World Health Organization, Geneva, Switzerland; Department of Infectious Diseases and Tropical Medicine, University Hospital, Ludwig-Maximilians University, Munich, Germany; Mycobacteriology Unit, Department of Microbiology, Institute of Tropical Medicine, Antwerp, Belgium

**Keywords:** Buruli ulcer, *Mycobacterium ulcerans*, Epidemiology, Case finding, Endemicity, Ogoja, Cross River State, Nigeria

## Abstract

**Background:**

Buruli ulcer (BU), also known as *Mycobacterium ulcerans* disease, is the third most common mycobacterial disease worldwide. Although BU disease has been diagnosed among Nigerians in neighbouring West African countries, data on the burden of the disease in Nigeria itself are scanty. This study aimed to assess the magnitude and epidemiology of BU in the South South region of Nigeria.

**Methods:**

We conducted a cross-sectional survey in the Ogoja territory (comprising 31 communities). We undertook sensitisation programmes centred on BU in 10 of the communities. Participants were asked to identify community members with long-standing ulcers, who were then invited for evaluation. We also contacted traditional healers to refer their clients who had non-healing ulcers. All suspected cases had a full clinical evaluation and laboratory testing. Confirmed cases were given treatment in a referral hospital in the territory.

**Results:**

We diagnosed 41 clinical BU cases; 36 (87.8 %) of which were confirmed by quantitative polymerase chain reaction (qPCR). These 36 PCR-confirmed cases were diagnosed in a total population of 192,169 inhabitants. Therefore, the estimated crude prevalence of BU was 18.7 per 100,000 population, varying from 6.0 to 41.4 per 100,000 in the districts surveyed. The majority (66.7 %) of the cases were females. About 92 % of the BU lesions were located on the patients’ extremities. No differences were observed between the sexes in terms of the location of the lesions. The age of the patients ranged from four to 60 years, with a median age of 17 years. All 35 (100 %) patients who consented to treatment completed chemotherapy as prescribed. Of the treated cases, 29 (82.9 %) needed and received surgery. All cases healed, but 29 (82.9 %) had some limitations in movement. Healing with limitations in movement occurred in 18/19 (94.7 %) and 8/10 (80.0 %) of patients with lesions >15 cm (Category III) and 6–15 cm in diameter (Category II), respectively. The median duration of treatment was 130 (87–164) days for children and 98 (56–134) days for adults (*p* = 0.15).

**Conclusions:**

In Nigeria, BU is endemic but its severity is underestimated—at least in the study setting. There is a need to identify and map BU endemic regions in Nigeria. A comprehensive BU control programme is also urgently needed.

**Electronic supplementary material:**

The online version of this article (doi:10.1186/s40249-016-0119-8) contains supplementary material, which is available to authorized users.

## Multilingual abstracts

Please see Additional file 1 for translations of the abstract into the six official working languages of the United Nations.

## Background

Buruli ulcer (BU), a neglected tropical disease, is caused by *Mycobacterium ulcerans*. Globally, it is the third most common mycobacteriosis after tuberculosis and leprosy [1]. The infection leads to the destruction of skin and soft tissue, presenting as large ulcers usually on the limbs. Patients who are not treated early can suffer permanent disfigurement and functional disability [1, 2]. Buruli ulcer has been reported in 33 countries in Africa, the Americas, Asia and the Western Pacific. Most cases occur in tropical and subtropical regions, except for in Australia, China and Japan [1, 2]. In West and Central Africa, Benin, Cameroon, Côte d’Ivoire, Democratic Republic of Congo (DRC) and Ghana have reported the majority of cases [3–7].

The exact mode of transmission of *M. ulcerans* is still unknown [1, 2]. Epidemiological studies have shown that BU is commonly found in populations living near rivers, swamps and wetlands [1, 2, 8]. In several instances, local environmental events, such as deforestation, flooding and building of dams, or agricultural activities such as irrigation, have been associated with the emergence of BU [1, 8]. The risk factors for BU that have been repeatedly identified include proximity to stagnant or slow-flowing bodies of water, poor wound care and not wearing protective clothing (e.g., long-sleeved shirts or shoes) [1, 8]. The probable role of aquatic insects as reservoirs and vectors of *M. ulcerans* has been proposed, but is still being debated [1, 8, 9].

Although Nigeria is surrounded by countries with a high endemicity of BU disease, only few cases have been reported to date. In 1967, four BU cases were first reported from Benue, Nigeria [10]. This was followed by 24 cases reported from Ibadan, in Oyo state, in 1976 [11]. Based on unofficial reports from some states in Southern Nigeria, the World Health Organization (WHO) carried out a rapid assessment in 2006, finding 14 cases with clinically-suspected BU [12]. In addition, a recent report mentioned that nine *M. ulcerans* strains were isolated from patients living in Oyo, Anambra, Cross River, Enugu, Ebonyi and Ogun states, during 2006 and 2012 [13]. Therefore, to date, 51 cases of BU have been reported from Nigeria. However, given the high number of BU patients from Nigeria being treated in neighbouring countries, such as Benin and Cameroon [14, 15], there seems to be a discrepancy, likely due to underreporting and inadequate public health structures to diagnose and treat the disease in Nigeria.

Furthermore, the WHO reports that there is evidence that BU disease is gradually increasing in incidence and that its geographic range is also increasing [16]. Given that the disease can suddenly appear in a new area which has previously been disease-free, especially in poor rural communities in Sub-Saharan Africa and other developing countries, basic data to help plan effective BU control activities are urgently needed, but lacking [1, 16]. The main objective of this pilot project was to ascertain the extent of BU disease in rural communities of Nigeria and provide an evidence base for a systematic national response. The specific objectives were to assess the minimum prevalence of BU and to determine the epidemiologic characteristics of BU in Ogoja territory, the target endemic region of the project.

## Methods

### Study area

**Fig. 1:**
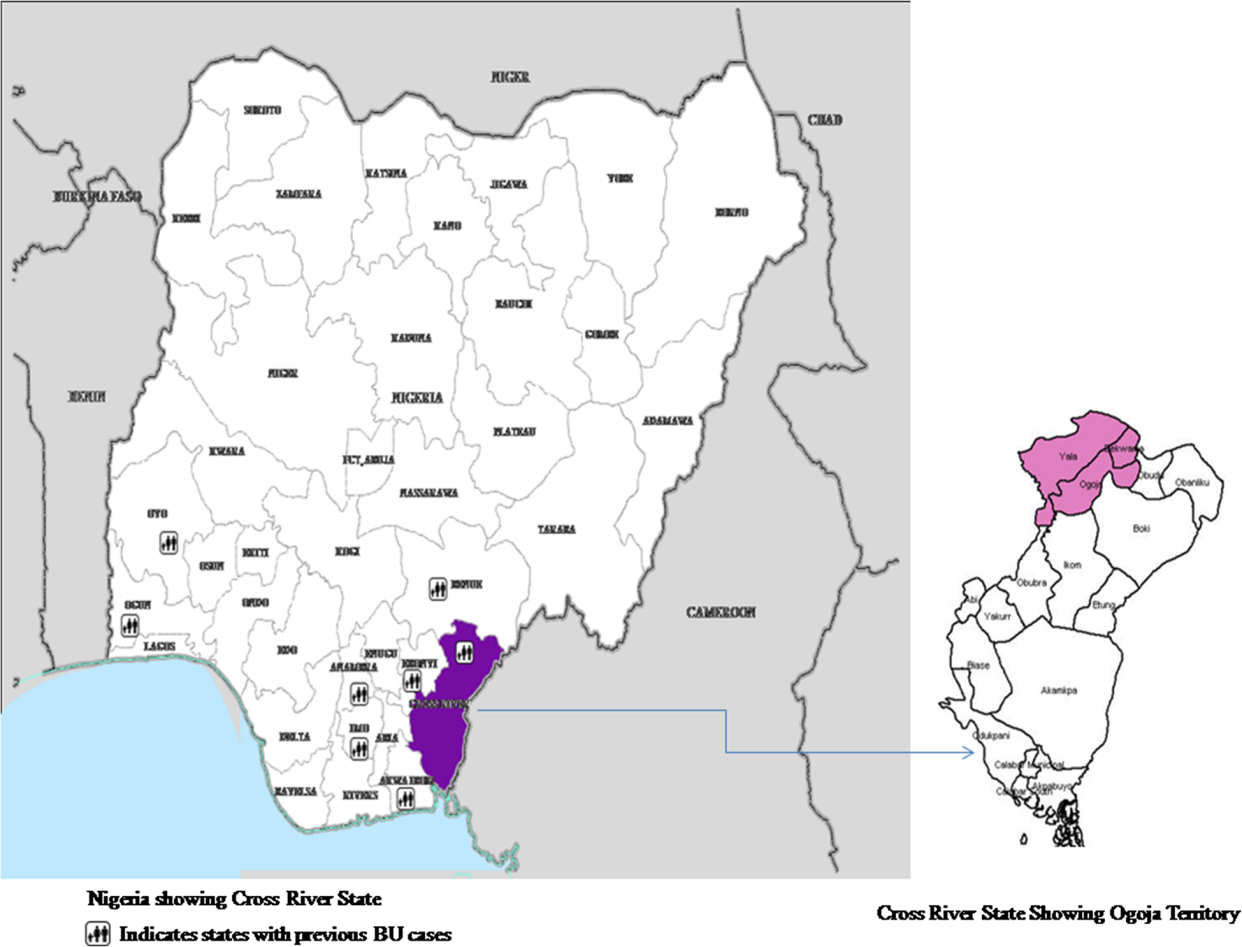
A map of Nigeria showing states where BU has been previously reported, highlighting the Cross River State and the three pilot project LGAs

The study was conducted in the Ogoja territory of Cross River State, Nigeria (see Fig. 1). The territory is a tropical rainforest belt surrounded by rivers and swamps. Cross River State derives its name from the Cross River, which transcends almost the entire length of the state and empties into the Atlantic (covering an area of 39,000 km^2^). The majority of the state’s inhabitants utilise the Cross River as source of drinking water, and most engage in farming of rice, yam and cassava. Three Local Government Areas (LGAs), i.e. Ogoja, Yala and Bekwarra, which previously constituted Ogoja territory, were selected as the study sites due to prior notification of BU in the region [12]. There are 31 communities in the three LGAs. Ten communities, which constitute about one third of the population of the three LGAs, were selected for the survey through simple random sampling, covering an estimated population of 192,169 people [17]. The study communities have 12 primary health centres and four secondary health care facilities, including the St Benedict Tuberculosis and Leprosy Rehabilitation Hospital Ogoja (SBHO), which is a private not-for-profit (faith-based) hospital that serves as a referral centre for tuberculosis and leprosy control in the whole of Ogoja.

### Study design

This was a community-based cross-sectional survey carried out between May 2012 and April 2013. It was conducted in two phases: a preparatory, and a case-finding and management phase. During the first three weeks of the project (May 2012), advocacy visits were held with the community leaders and health authorities, and their approval for the study was obtained. Project staff members in the facilities were trained on identification of BU suspects, sample collection, preparation and transportation. General health workers and doctors working in the peripheral health facilities in the three study LGAs were trained on BU symptoms recognition and appropriate referral.

The case-finding and management phase involved intensive advocacy, communication and social mobilisation. Due to logistical problems, it was not possible to undertake a door-to-door survey. However, outreach/sensitisation activities took the form of village hall/square meetings with the whole community. Community leaders were informed of the intention to hold outreach programmes in their community, a date was chosen for the event, community members were instructed to gather at the village hall/square, and finally the community sensitisation programme was undertaken. This was done monthly in all study communities. During the sensitisation activities, the communities were informed about the free BU treatment opportunities at the SBHO. Case finding formed part of the advocacy, communication and social mobilisation activities. Screening for BU disease was done onsite and persons clinically suspected were referred to the SBHO for evaluation, free laboratory investigation and treatment. In addition, traditional healers in each community were interviewed about managing individuals with chronic ulcers that have failed to heal. They were also trained to refer such cases for evaluation at the SBHO. Individuals suspected to have BU disease were interviewed about whether they knew other people who had similar lesions. Such cases were identified, evaluated and referred to the SBHO. Patients suspected of having BU disease that were referred to or presented at the SBHO had their diagnoses clinically validated by trained physicians to ensure that they were consistent with the WHO clinical case definitions [18, 19], and subsequently had other management strategies initiated.

### Laboratory confirmation

Laboratory confirmation of clinical BU disease cases involved taking swabs from ulcerative lesions and fine needle aspirates from pre-ulcerative lesions, followed by laboratory testing (microscopy and/or molecular biology) using the WHO guidelines [18]. Samples were also sent to the Institute of Tropical Medicine Antwerp and the Division of Infectious Diseases and Tropical Medicine, Medical Centre of The University of Munich, where quantitative polymerase chain reaction (qPCR) was performed for the detection of *M. ulcerans*.

### Clinical care and management

Case management was carried out according to the WHO recommendations [19]. Each patient received an eight-week drug treatment (chemotherapy) using the standard regimen of rifampicin and streptomycin. Wound dressing for ulcerated cases was an important component of clinical management. For each patient, this process continued until the wound had either healed completely or adequately granulated for skin grafting. Surgery was part of the ulcer management, as some of the cases required surgical interventions such as debridement, skin grafting and/or amputation. Physiotherapy was provided to those who had contractures and limitation in movement.

### Statistical analysis

The data were recorded on a standardised BU report form, double-entered into a Microsoft Excel (Microsoft Office Inc for Windows, USA) database and analysed using Epi Info version 3.4.1 (Centers for Diseases Control and Prevention, Atlanta, GA, USA). Continuous variables were summarised as medians (interquartile ranges), and categorical variables as counts and percentages. The Fisher’s exact or chi-square test was used to compare categorical proportions, and the Student’s *t*-test or Mann–Whitney *U* test was used in the case of continuous variables. The significance level was set at 5 %.

### Ethics statement

Ethical approval for this study was obtained from the Ethical Advisory Board of the German Tuberculosis and Leprosy Relief Association. Permission was also obtained from the management of the SBHO. All patients or their legal guardians (for minors) gave written informed consent for all diagnostic and treatment purposes, as well as for the publication of clinical photographs. All BU cases part of the study were treated at no financial costs to them.

## Results

### Laboratory confirmation of cases

In the one-year period of the project, 41 patients with clinical BU lesions at different stages of development were identified. Of these, 36 (87.8 %) were confirmed by qPCR. Of the cases not confirmed in the laboratory, four (9.8 %) tested negative by qPCR and one (2.4 %) didn’t have samples collected. In addition, 45.2 % (14/31) of the clinically confirmed BU cases who had a smear examination also tested positive using Ziehl-Neelsen staining—all of whom were also positive for BU using the qPCR method. We restricted our subsequent report to the 36 cases who were positive for BU by qPCR.

### Clinical presentation

**Fig. 2:**
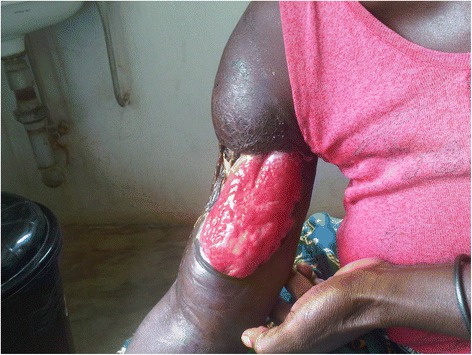
A typical Buruli ulcer on the upper limb of an adult male identified during the study

**Fig. 3:**
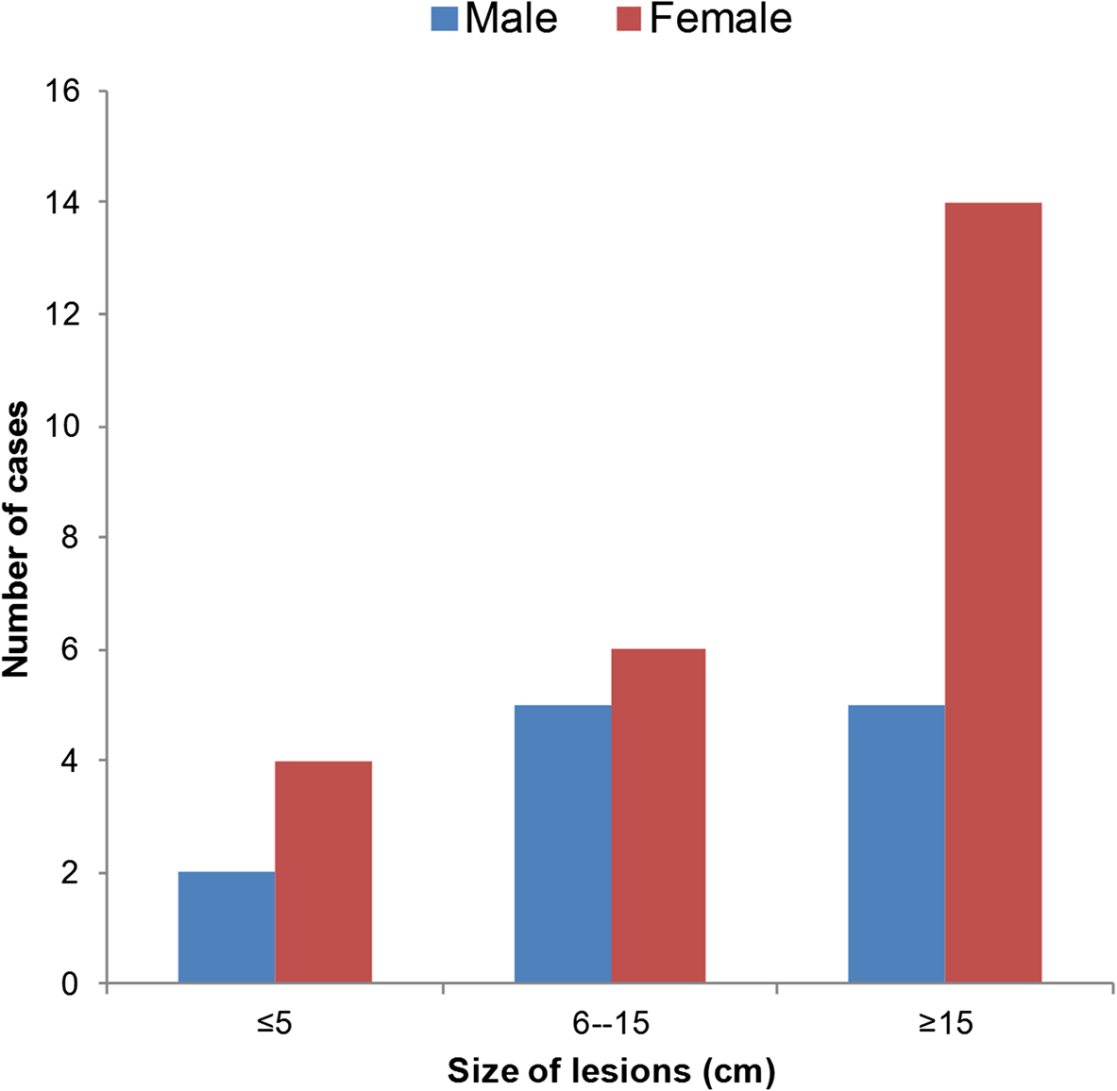
Distribution and sizes of Buruli ulcer lesions, according to patients’ sex

All 36 (100 %) patients were newly diagnosed BU disease cases. About 88.9 % of the lesions were at the ulcerative stage (see Fig. 2) and 11.1 % were at the pre-ulcerative stage. Lesions occurring at both the ulcerative and pre-ulcerative stages were seen in 2.8 % of the patients. Table 1 shows the clinico-epidemiological features of the cases. The locations of the lesions were variable: 19 (52.8 %) had lesions on the lower limb, 14 (38.9 %) had lesions on the upper limb and three (8.3 %) had lesions on the trunk. More than half (52.8 %; 19) of the patients presented advanced ulcer lesions that were more than 15 cm in diameter (Category III lesions) (see Fig. 3), the majority of which occurred in females (58.3 % vs. 41.7 %; *p* = 0.35); while 11 (30.6 %) had lesions that were 6–15 cm in diameter (Category II lesions). In addition, 24 (67 %) had functional limitations in movement, with these limitations most significantly occurring (58.3 %—14 patients) in patients with lesions on the lower limb.

**Table 1:** Clinico-epidemiological features of Buruli ulcer cases in Ogoja territory, May 2012 to April 2013

Characteristics		Buruli ulcer case n (%)
Sex	Female	24 (66.7)
	Male	12 (33.3)
Age group	≤15 years	15 (41.7)
	>15 years	21 (58.3)
Classification of cases	New case	36 (100)
	Recurrent	0 (0)
Clinical form	Plaque	4 (11.1)
	Ulcer	31 (86.1)
	Ulcer and oedema	1 (2.8)
Site of lesion	Upper limb	14 (38.9)
	Lower limb	19 (52.8)
	Trunk	3 (8.3)
Size of lesion	Category I	6 (16.7)
	Category II	11 (30.6)
	Category III	19 (52.8)
Limitation of movement?	Yes	24 (66.7)
	No	12 (33.3)

### Epidemiological features

Using the identified active lesions (pre-ulcerative or ulcerative) and the populations of the study communities, we estimated that the crude minimum prevalence rate was 18.7 per 100, 000. Table 2 shows the minimum prevalence and distribution of BU disease cases according to the size of the population where the survey was conducted. Ogoja LGA had the highest prevalence rate of 41.4 per 100,000 population. Of the PCR-confirmed BU cases, 24 (66.7 %) were female and 12 (33.3 %) were male. The ages of the patients ranged from four to 60 years (median: 17 years, mode: 20 years). Overall, 15 (41.7 %) were children (≤15 years). No statistical difference was observed in terms of sex among patients who were children (≤15 years) compared with adults >15 years old (Fisher’s exact test: *p* = 0.14) (see Table 1).

**Table 2:** Prevalence of Buruli ulcer in the Ogoja territory, May 2012 to April 2013

LGA	Number of communities	Population	Number of BU cases	Crude prevalence per 100,000
Bekwarra	1	41,623	3	7.2
Ogoja	7	67,614	28	41.4
Yala	2	82,932	5	6.0
Total population	10	192,169	36	18.7

The distribution of lesions on the limbs and trunk did not differ among the sexes (*χ*^2^ = 1.4; *p* = 0.24). Among females, lesions occurred equally (45.8 %) on the upper limb and the lower limb, while the remaining 8.4 % occurred on the trunk. Among males, 66.7 % of the lesions occurred on the lower limb, 25.0 % on the upper limb and 8.3 % on the trunk. The distribution of the lesions also did not differ by age group (Fisher’s exact test: *p* = 0.455). Among children, 53.3 % of the lesions occurred on the lower limb, 40 % on the upper limb and 6.7 % on the trunk. Among adults, 52.4 % of lesions occurred on the lower limb, 38.1 % on the upper limb and 9.5 % at the trunk.

### Case management

**Fig. 4:**
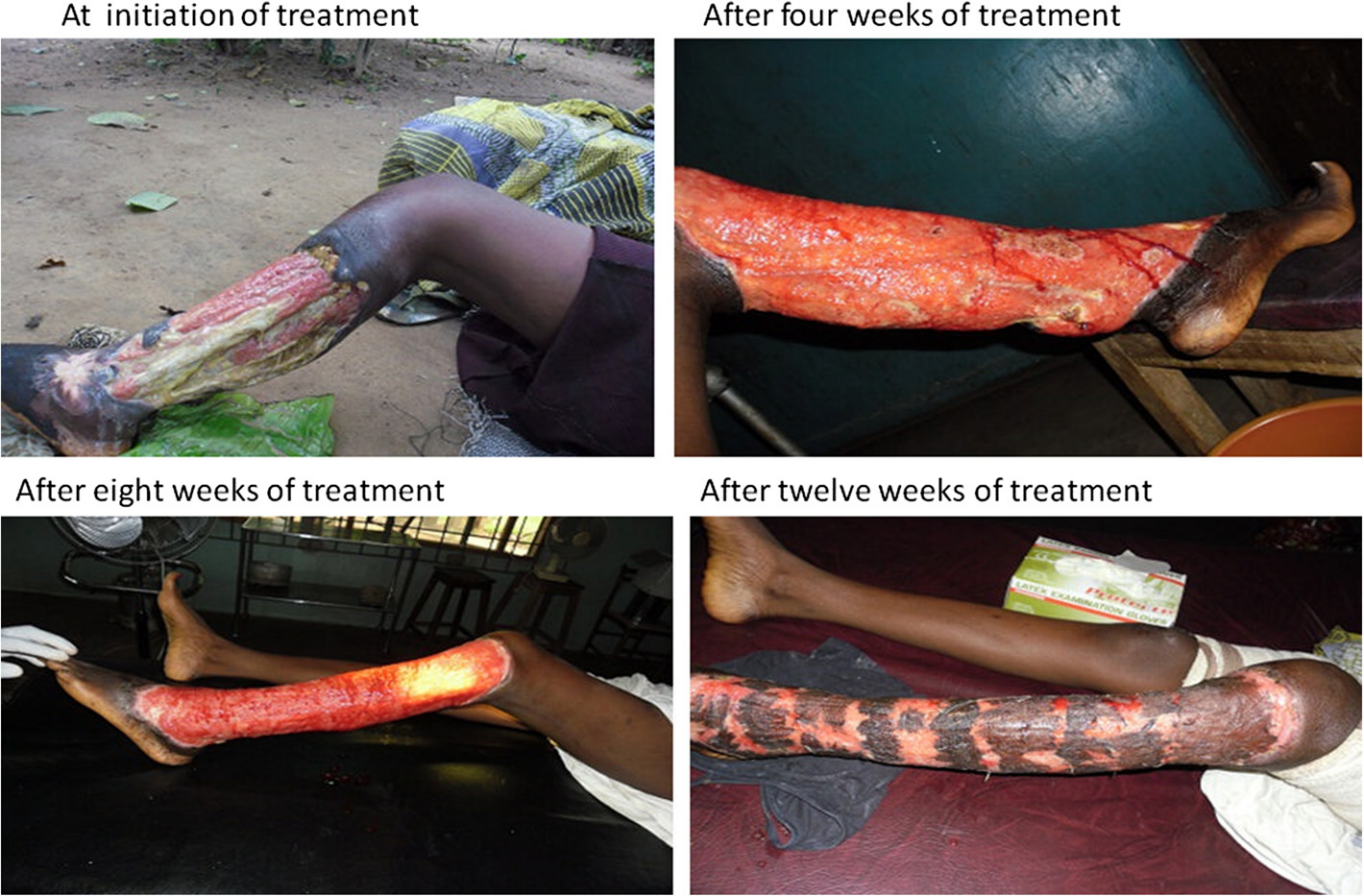
Progressive healing of a Buruli ulcer patient who was given treatment

**Fig. 5:**
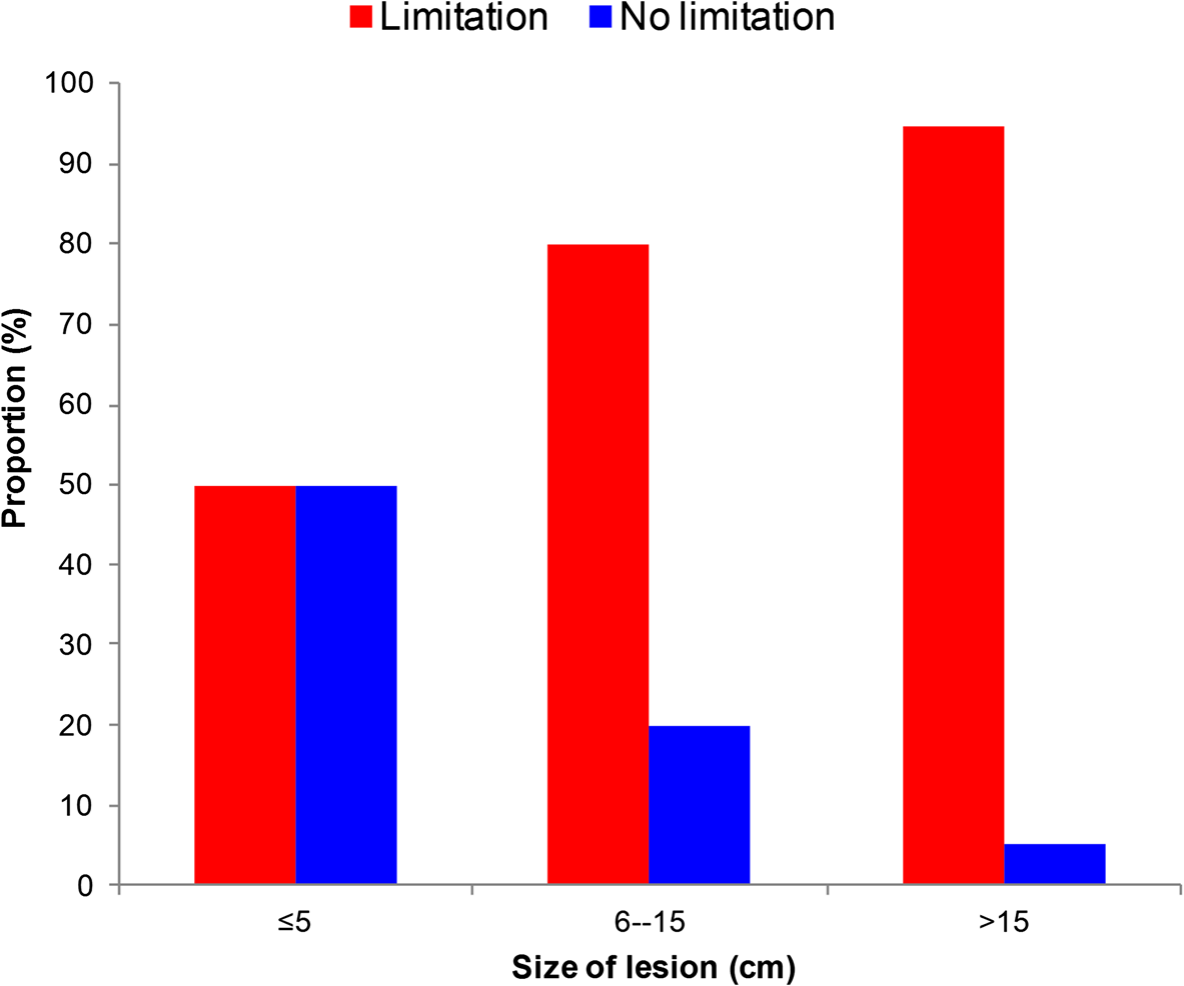
Proportion of healed Buruli ulcer cases with or without limitation in movement, according to size of the lesion

Of the cases diagnosed, 35 were treated with antibiotics (oral rifampicin and streptomycin injection) for eight weeks, while one patient declined treatment. Of the treated cases, 29 (82.9 %) needed and received surgery. All 35 treated cases completed treatment and were discharged. Figure 4 shows the progression of one case’s healing process. Of the total number of cases, 29 (82.9 %) healed with some limitations in movement, while six (17.1 %) cases healed with no limitation in movement. Healing with limitations in movement occurred in 18/19 (94.7 %), 8/10 (80.0 %) and 3/6 (50 %) of patients with Category III (>15 cm), Category II (6–15 cm) and Category I (≤5 cm) lesions, respectively (see Fig. 5). All the cases that healed with limitations in joint movement received physiotherapy, with some improvements observed. Overall, the treatment duration (including hospitalisation/dressings) ranged from 56 to 244 (median of 135) days. Patients with Category III, II and I lesions had median durations of treatment of 120, 116 and 83, days, respectively (*p* = 0.37). The median duration of treatment was 130 (87–164) days for children and 98 (56–134) days for adults (*p* = 0.15).

## Discussion

The overall crude prevalence rate of BU of 18.7 per 100,000 in the study communities (and the much higher rate of 41.4 per 100,000 in Ogoja) indicates that BU is prevalent in the region, and particularly in Ogoja territory. Before this pilot project, a cumulative total of 51 BU cases have been reported from Nigeria [15]—only three of these were from Ogoja LGA [12]. This suggests that in endemic communities in Nigeria, the number of BU cases is grossly underestimated. Therefore, there is an urgent need to step up BU case finding in communities where standard reporting has previously identified at least one BU case. The crude prevalence rate of BU found in this study is comparable to the 21 and 30 per 100,000 cases found in nationwide surveys in Ghana and Cote d’Ivoire, respectively [6, 7]. However, our finding was lower than 184, 205 and 330 per 100,000 population reported in a district in Benin, Cameroon and DRC, respectively [5, 14, 20, 21]. In our current effort, we have probably missed several prevalent BU cases that would have been identified if more active case-finding activities were implemented. Although BU is a notifiable disease in Nigeria, there have not been any deliberate efforts to engage the affected communities to identify and treat the disease. One of the commonly held notions by heath policymakers in Nigeria is that BU disease is not endemic in the country. The findings of this study disagree with this belief and justify the need for the institution of deliberate efforts to identify and control BU.

The median age of cases in this study was comparable to patients aged 15.5 years, as reported from Cameroon [20]. It was lower than the median age observed in Ghana (25 years) and DRC (27 years), but higher than the median age of 12 years reported in Benin [5, 6, 22]. Overall, our study agrees with previous studies that found that the highest rate of the disease is observed among children <15 years of age in disease-endemic communities [5–7, 21, 22]. In addition, we found that >60 % of cases occurred in females. Previous studies didn’t observe any statistical differences in the occurrence of cases among the sexes [3, 6, 14, 22]. This difference may be attributable to a higher proportion of females participating in sensitisation and project activities in the communities, and therefore those with lesions had a higher likelihood to be screened and assessed, as census data suggest no female sex dominance in the population [17].

About 92 % of the BU cases had lesions on their extremities in our study. This is consistent with observations in Ghana, Côte d’Ivoire and DRC, which found that more than 90 % of the lesions occur on the limbs [5–7]. Females had an equal number of lesions on the upper and lower limbs, while males predominantly had lesions on the lower limbs. The reason/s for this difference is not clear, but this finding is consistent with the findings of Barker [23]. However, recent surveys have observed no differences among the sexes in the distribution of lesions [3, 6, 14].

The most common clinical presentation was an ulcerative lesion, which occurred in almost 90 % of the cases. This agrees with studies conducted in the DRC, Cote d’Ivoire and Cameroon [4, 5, 7, 14]; while the proportion of patients with ulcerative lesions was lower in previous surveys undertaken in Ghana and Benin, which recorded proportions of ulcerative lesions ranging from 48 % to 58 % [3, 6, 21]. Furthermore, two-thirds (67 %) of the cases had functional limitations of joint movement at presentation. This contrasts with previous findings which show that this occurs in less than one third of cases in other African endemic regions [5, 20]. This may be because more than half of the BU cases in this study had Category III lesions and were prone to complications. Moreover, late presentation due to a lack of BU control activities in Nigeria may have contributed to BU-related complications, resulting in a high proportion of cases with limitations of joint movement. Previous reviews have suggested an equal distribution of Category I, II, and III lesions [1]. The higher proportion of Category III lesions observed in this study may partly be explained by the study design, which focused on finding individuals with chronic ulcers in the community.

In addition, the identification of patients with more advanced ulcerative lesions may have allowed easier quality sample collection, which contributed to the high rate of laboratory confirmation of clinical cases. Most studies in other endemic regions in Africa had rates of laboratory-confirmed BU cases ranging from 30 % to 74 % [24–27]. Thus, the high rate of laboratory-confirmed cases in this survey may reflect a higher endemicity of active BU cases in the study communities than was previously observed. Moreover, given that only relatively few cases had Category I and II lesions, future surveys should target households, in order to increase the detection, diagnosis and treatment of individuals with early BU lesions in the community. Also, project staff and general health workers should be trained to identify early lesions of BU (i.e. oedema, nodules, etc.).

All treated BU cases completed chemotherapy and none were lost to follow-up. This reflects the effectiveness of the project in retaining cases and is comparable to other studies that showed high rates of successful outcomes of treated BU cases [24, 27]. The free treatment and socio-economic support provided by the project to the patients may have contributed to the success of the disease management. A previous study showed that even accessing highly-subsidised BU treatment programmes was challenging for patients, leading to many of them abandoning biomedical treatment altogether [28]. Future BU control projects in Nigeria will benefit from adopting the lessons learnt from the successes of this project.

A gap in our study is the lack of data on the HIV status of patients. In any case, there is little evidence from literature that there is an association between HIV and BU [29]. Secondly, unlike other studies in BU endemic settings [5–7], we adopted a strategy that combined different methods to detect the cases; a door-to-door survey may have yielded more cases.

## Conclusion

In conclusion, this study has a number of strengths, including that it confirmed the endemicity of BU in the study population. We also identified—for the first time—more cases of clinical and laboratory-confirmed BU in Nigeria compared to any period in the past, as previous cases have not been sought. In addition, all treated cases completed chemotherapy. This study shows that BU disease is endemic in Ogoja territory of Cross River State and that the burden of the disease is likely to have been grossly underestimated in Nigeria. In all areas where BU has been reported in the last decade, the burden of the disease may be substantially higher than currently documented through the routine reporting system. We recommend that a phased systematic nationwide response based on lessons learnt from the pilot BU control programme be established—this may be integrated with National Tuberculosis and Leprosy Control Programme. We also believe that the identification and mapping of BU endemic regions in Nigeria should be strengthened.

## Additional file

Additional file 1: Multilingual abstracts in the six official working languages of the United Nations. (PDF 329 kb)

## Abbreviations

BU: buruli ulcer
DRC: Democratic Republic of Congo
LGA: local government area
qPCR: quantitative polymerase chain reaction
SBHO: St Benedict tuberculosis and leprosy rehabilitation hospital Ogoja
WHO: World Health Organization
