# Supplementary material for: Buruli ulcer in Nigeria: results of a pilot case study in three rural districts
Source: Infect Dis Poverty. 2016 Apr 22;5:39. doi: 10.1186/s40249-016-0119-8 (PMC4841952; doi:10.1186/s40249-016-0119-8)
Supplement: Additional file 1: — Multilingual abstracts in the six official working languages of the United Nations. (PDF 329 kb) [file 40249_2016_119_MOESM1_ESM.pdf]

Translation of the abstract into the five official working languages of the United Nations

قرحة بورولي في نيجيريا: نتائج دراسة أولية في ثلاث مناطق ريفية

Kingsley N. Ukwaja, Anthony O. Meka, Alphonsus Chukwuka, Kingsley B. Asiedu, Kristina L. Huber, Miriam Eddyani, Joseph N. Chukwu, Moses C. Anyim, Charles C. Nwafor, Daniel C. Oshi, Nelson O. Madichie, Ngozi Ekeke, Martin Njoku, Kentigern Ntana

الملخص

خلفية: قرحة بورولي، والمعروفة أيضًا باسم مرض القرحة المتقطرة، هي ثالث الأمراض الفطرية شيوعًا في العالم. وعلى الرغم من أن قرحة بورولي قد تم تشخيصها بين النيجيريين في بلدان غرب أفريقيا، إلا أن البيانات المتوفرة عن عبء المرض في نيجيريا هزيلة. تهدف هذه الدراسة إلى تقييم حجم ووباء قرحة بورولي في جنوب الإقليم الجنوبي في نيجيريا.

المنهج: أجرينا دراسة مقطعية في إقليم أوغوجا (والتي تضم 31 مجتمعًا). ووضعنا برامج للتوعية تركز على قرحة بورولي في 10 من هذه المجتمعات. طلب من المشاركين تحديد أفراد المجتمع الذين يعانون من قرحة طويلة الأمد لتتم دعوتهم بعدها للتقييم. كما تواصلنا مع المعالجين التقليديين ليحيلونا إلى مرضاهم المصابين بقرحة لا يرجى الشفاء منها. خضعت جميع الحالات المشتبه فيها إلى تقييم طبي وفحوصات مخبرية، وتم إخضاع الحالات المؤكدة للعلاج في المستشفيات الموجودة في المنطقة.

النتائج: قمنا بتشخيص 41 حالة قرحة بورولي سريريًا: 36 (87.8%) تم التأكد من إصابتها من خلال تفاعل البوليميراز المتسلسل الكمي اللحظي (qPCR) وتم تشخيص هذه الحالات المؤكدة في عدد بلغ 192169 شخصًا من مجموع السكان. ومن هنا، يقدر انتشار قرحة بورولي بـ 18.7 لكل 100000 نسمة، تتراوح بين 6.0 إلى 41.4 لكل 100000 في المناطق التي شملتها الدراسة. وكانت غالبية الحالات (66.7%) من الإناث. ووجد ما يقرب من 92% من آفات قرحة بورولي على أطراف أجسام المرضى، ولم تتم ملاحظة أي فروق بين الجنسين من حيث موقع آفات المرض. وتروحت أعمار المرضى بين 4 إلى 60 سنة، في حين يبلغ متوسط الأعمار 17 سنة. جميع المرضى الـ 35 (100%) من الذين وافقوا على الخضوع للعلاج الكيماوي أنهوه على النحو الذي تم وصفه. ومن بين الحالات التي تم علاجها، تطلبت وخضعت 29 (82.9%) حالة لعملية جراحية. وعلى الرغم من شفاء جميع الحالات، إلا أن 29 منها واجهت بعض الصعوبات في الحركة. وقعت هذه الصعوبات في 19/18 (94.7%) و10/8 (80.0%) من المرضى الذين يعانون من الآفات >15 سم (الفئة 3) و 6-15 سم قطريًا (الفئة 2)، على التوالي. وبلغ متوسط مدة العلاج 130 (87-164) يومًا للأطفال و 98 (56-134) يومًا للكبار (المدة =0.15).

النتيجة: تستوطن قرحة بورولي في نيجيريا، إلا أن خطورتها يستهان بها - في حالة هذه الدراسة على الأقل. من الضروري تحديد ورسم خريطة للمناطق التي تنتشر فيها قرحة بورولي في نيجيريا، كما أن هناك حاجة ماسة إلى برنامج شامل للسيطرة على هذه القرحة.

Translated from English version into Arabic by Shada Salameh, through

TRANSLATORS
WITHOUT BORDERS

尼日利亚布鲁里溃疡：三个农村地区试点案例研究结果

Kingsley N. Ukwaja, Anthony O. Meka, Alphonsus Chukwuka, Kingsley B. Asiedu, Kristina L. Huber, Miriam Eddyani, Joseph N. Chukwu, Moses C. Anyim, Charles C. Nwafor, Daniel C. Oshi, Nelson O. Madichie, Ngozi Ekeke, Martin Njoku, Kentigern Ntana

摘要

引言：布鲁里溃疡也称溃疡分枝杆菌病，是世界范围内最常见的排名第三的分枝杆菌疾病。虽然在尼日利亚周边的西非国家人群中确诊了布鲁里溃疡，但是在尼日利亚该疾病负担的数据仍然不足。本研究目的在

于评估尼日利亚南部地区布鲁里溃疡的流行程度和范围。

方法：我们在Ogoja区域（包含31个社区）进行横断面调查。在其中10个社区实施布鲁里溃疡的宣传工作。通过问询参与者来确认社区内是否有长期溃疡的人员，随后邀请这类人参加评估。并通过联系传统治疗师来寻找他们的客户中有未治愈溃疡的患者。所有的疑似病例均接受全面的临床评估和实验室诊断。 确诊病例在Ogoja区域的中心医院接受治疗。

结果：共诊断了 41 例临床病例，其中 36 例（87.8%）经定量 PCR（qPCR）确诊。经 qPCR 确诊的 36 例病例来自于当地 192 169 居民中。因此，在调查区域估计布鲁里溃疡的发病率为 18.7/10 万，范围从 6.0～41.4/10 万。病例大部分为女性（66.7%）。约 92%布鲁里溃疡病变位于患者四肢。关于病变的位置在性别上并无显著差异。患者年龄从 4 岁到 60 岁，中位数年龄为 17。所有同意接受治疗的 35 例病例均按照规定完成化疗，29 例病例(82.9%)需要并接受了手术治疗。所有病例均被治愈，但有 29 例(82.9%)出现活动受限。在病变>15 cm (Ⅲ类)和病变为 6–15 cm (Ⅱ类)患者中，活动受限恢复率分别为 18/19 (94.7%)和 8/10 (80.0%)。儿童和成人治疗期中位数分别为 130 (87～164) d 和 98 (56～134) d (P＞0.05)。

结论：布鲁里溃疡在尼日利亚流行，但是其严重程度被低估-------至少在本研究的区域是这样的。因此，有必要核实布鲁里溃疡在尼日利亚的流行区域和对该疾病进行疾病制图。同时急需开展针对布鲁里溃疡的综合控制项目。

Translated from English version into Chinese by Feng Xin-Yu, edited by Yang Pin, through

CHINESE CENTER FOR DISEASE CONTROL AND PREVENTION
NATIONAL INSTITUTE OF PARASITIC DISEASES

L'ulcère de Buruli au Nigeria : résultat d'une étude de cas pilote dans trois districts ruraux

Kingsley N. Ukwaja, Anthony O. Meka, Alphonsus Chukwuka, Kingsley B. Asiedu, Kristina L. Huber, Miriam Eddyani, Joseph N. Chukwu, Moses C. Anyim, Charles C. Nwafor, Daniel C. Oshi, Nelson O. Madichie, Ngozi Ekeke, Martin Njoku, Kentigern Ntana

Résumé

Contexte : L'ulcère de Buruli, causé par *Mycobacterium ulcerans*, est la troisième mycobactériose par ordre de fréquence dans le monde. Bien qu'il ait été diagnostiqué chez des Nigérians dans des pays limitrophes d'Afrique occidentale, le poids de la maladie au Nigeria même est mal connu. Cette étude avait pour but d'évaluer l'étendue et l'épidémiologie de l'ulcère de Buruli dans le sud du Nigeria.

Méthodes : Nous avons mené une étude transversale dans le Territoire d'Ogoja (31 villages) Nous avons entrepris des programmes de sensibilisation à l'ulcère de Buruli dans 10 de ces villages, en demandant aux participants d'identifier les membres de leur communauté qui présentaient des ulcères depuis longtemps. Les patients signalés ont ensuite été invités à une évaluation. Nous avons également contacté les guérisseurs traditionnels en leur demandant de nous adresser leurs clients porteurs d'ulcères qui ne guérissaient pas. Tous les cas suspects ont fait l'objet d'un bilan clinique complet et de tests en laboratoire. Les cas confirmés ont été traités dans un hôpital de référence sur le territoire.

Résultats : Nous avons diagnostiqué 41 cas d'ulcère de Buruli, dont 36 (87,8 %) ont été confirmés par une réaction en chaîne à la polymérase quantitative (RCPq). Ces 36 cas confirmés par la RCP ont été diagnostiqué dans une population totale de 192 169 habitants. La prévalence brute estimée de l'ulcère de Buruli serait donc de 18,7 cas par 100 000 habitants, avec des extrêmes de 6,0 et 41,6 pour 100 000 dans les districts étudiés. La plupart des

cas (66,7 %) étaient des fillettes ou des femmes. Près de 92 % des ulcères se trouvaient sur les extrémités. Nous n’avons observé aucune différence entre les sexes quant à la localisation des lésions. L’âge des patients allait de 4 à 60 ans, avec une médiane à 17 ans. Les 35 patients qui ont consenti au traitement ont tous mené la chimiothérapie à terme dans le respect des prescriptions (100 %). Sur les cas traités, 29 (82,9 %) ont eu besoin de chirurgie et ont été opérés. Tous les ulcères ont cicatrisé, mais en laissant des limitations de la mobilité dans 29 cas (82,9 %). Cette limitation de la mobilité après cicatrisation concernait 18 (94,7 %) des 19 patients porteurs de lésions de plus de 15 cm de diamètre (catégorie III) et 8 (80,0 %) des 10 patients dont les lésions mesuraient entre 6 et 15 cm (catégorie II). La durée médiane du traitement était de 130 (87-164) jours pour les enfants et 98 (56-134) jours pour les adultes (p = 0,15).

Conclusions : L’ulcère de Buruli est endémique au Nigeria mais sa sévérité s’avère sous-estimée, tout au moins dans le cadre de l’étude. Les régions d’endémie dans le pays doivent être identifiées et cartographiées, et il est urgent de mettre en place un programme complet de lutte contre la maladie.

Translated from English version into French by eric ragu, through

TRANSLATORS
WITHOUT BORDERS

Язва Бурули в Нигерии: Результаты анализа клинических случаев в трех сельских районах

Кингсли Н. Уквайа, Энтони О. Мека, Альфонсус Чуквука, Кингсли Б. Асьеду, Кристина Л. Губер, Мириам Эддиани, Джозеф Н. Чукву, Мозес С. Аньим, Чарльз С. Нвафор, Данель С. Оши, Нельсон О. Мадикье, Нгози Экеке, Мартин Ньоку, Кентигенрн Нтана

РЕФЕРАТ

История вопроса: Язва Бурули (BU), также известная как *Mycobacterium ulcerans*, является третьей в списке наиболее распространенных в мире микобактериальных инфекций. Хотя язва Бурули диагностировалась у нигерийцев в соседних западноафриканских странах, не имеется достаточно данных по уровню заболеваемости в самой Нигерии. Целью данного исследования является оценка распространенности и эпидемиологии язвы Бурули в южных регионах Нигерии.

Методы: Нами проведено перекрёстное исследование в районе Огойа (включающем 31 общину). Были осуществлены ознакомительные программы, концентрирующие внимание на язве Бурули в 10 из этих общин. Участникам было предложено идентифицировать членов общин с застарелыми язвами, которых затем пригласили для обследования. Мы также связались с целителями и попросили их сообщить о пациентах с незаживающими язвами. Все подозрительные случаи были подвергнуты полной клинической оценке и лабораторным исследованиям. При подтверждении диагноза пациенты направлялись на лечение в районные лечебно-диагностические центры.

Результаты: Было диагностировано 41 клинический случай язвы Бурули; 36 (87,8%) из которых были подтверждены с помощью количественной полимеразной цепной реакции (qPCR). Эти 36 подтвержденных с помощью полимеразной цепной реакции случаев были выявлены в популяции из 192 169 жителей. Следовательно, оцененное приближенное значение распространенности язвы Бурули составило 18,7 на 100 000 населения, в диапазоне от 6,0 до 41,4 на 100 000 населения в зависимости от района обследования.

Большинство случаев (66,7%) составили женщины. Около 92% поражений язвой Бурули располагались на конечностях пациентов. Не было отмечено разницы между полами в отношении расположения повреждений. Возраст пациентов составил от четырех до 60 лет, медианный возраст 17 лет. Все 35 (100%) пациентов, согласившиеся на лечение, прошли курс химиотерапии. 29 (82.9%) из прошедших лечение пациентов нуждались в хирургической операции и получили ее. Во всех случаях было достигнуто выздоровление, но у 29 пациентов (82.9%) остались ограничения движения. Излечение с ограничениями в движениях зарегистрировано у 18/19 (94,7%) и 8/10 (80,0%) пациентов с очагом повреждений >15 см (категория III) и 6–15 см в диметре (категория II), соответственно. Средняя продолжительность лечения составила 130 (87–164) дней у детей и 98 (56–134) дней у взрослых (p = 0.15).

Выводы: Язва Бурули является эндемичным заболеванием в Нигерии, но его серьезность недооценивается, по крайней мере в условиях данного исследования. Необходимо определить и составить карту эндемичных по язве Бурули регионов Нигерии. Необходимо также срочно разработать всеобъемлющую программу по борьбе с язвой Бурули.

Translated from English version into Russian by Alena Hrybouskaya, through

TRANSLATORS WITHOUT BORDERS

La úlcera de Buruli en Nigeria: Resultados de un estudio piloto de casos en tres distritos rurales

Kingsley N. Ukwaja, Anthony O. Meka, Alphonsus Chukwuka, Kingsley B. Asiedu, Kristina L. Huber, Miriam Eddyani, Joseph N. Chukwu, Moses C. Anyim, Charles C. Nwafor, Daniel C. Oshi, Nelson O. Madichie, Ngozi Ekeke, Martin Njoku, Kentigern Ntana

RESUMEN

Introducción: La úlcera de Buruli (UB), conocida también como enfermedad por *Mycobacterium ulcerans*, es la tercera enfermedad producida por micobacterias más frecuente en todo el mundo. Aunque la UB ha sido diagnosticada entre nigerianos en países vecinos de África occidental, los datos relativos a la carga de la enfermedad en Nigeria son escasos. Este estudio tuvo como finalidad evaluar la magnitud y la epidemiología de la UB en la región del sur de Nigeria.

Métodos: Realizamos una encuesta transversal en el territorio de Ojoga (que comprende 31 comunidades). Emprendimos programas de sensibilización centrados en la UB en 10 de estas comunidades. Pedimos a los participantes que identificaran a miembros de su comunidad con úlceras de larga evolución, los cuales fueron invitados a someterse a una evaluación. También nos pusimos en contacto con curanderos tradicionales para derivar a sus clientes con úlceras difíciles de curar. A todos los casos sospechosos se les efectuó una evaluación clínica completa y pruebas analíticas. A los casos confirmados se les dispensó tratamiento en alguno de los hospitales de referencia del territorio.

Resultados: Diagnosticamos 41 casos de UV clínica, 36 (87,8 %) de los cuales fueron confirmados mediante una prueba cuantitativa de la reacción en cadena de la polimerasa (PCRc). Estos 36 casos confirmados por PCR fueron diagnosticados entre una población total de 192 169 habitantes. Por consiguiente, se estimó una prevalencia bruta de UB de 18,7 casos por 100 000 habitantes, que variaba entre 6,0 y 41,4 casos por 100 000 habitantes según el

distrito analizado. La mayoría (66,7 %) de cases fueron mujeres. Alrededor de un 92 % de las lesiones de UB de los pacientes estaban situadas en las extremidades. No se observaron diferencias entre sexos en cuanto a la localización de las lesiones. El rango de edad de los pacientes fue de 4 a 60 años, con una mediana de edad de 17 años. Los 35 pacientes (100%) que consintieron recibir tratamiento, completaron la quimioterapia prescrita. De los casos tratados, 29 (82,9 %) precisaron y se sometieron a una intervención quirúrgica. Todos los casos se curaron, pero 29 (82,9 %) presentaron algunas limitaciones de movimiento. Se observó curación con limitaciones de movimiento en 18/19 (94,7 %) y 8/10 (80,0 %) de los pacientes con lesiones > 15 cm (Categoría III) y 6–15 cm de diámetro (Categoría II), respectivamente. La duración mediana del tratamiento fue de 130 (87–164) días en los niños y de 98 (56–134) días en los adultos (p = 0,15).

Conclusiones: En Nigeria, la UB es endémica, pero su gravedad está infraestimada, al menos en el entorno en el que se llevó a cabo este estudio. Es necesario identificar y cartografiar las regiones nigerianas en las que la UB es endémica. También es urgente realizar un programa integral de control de la UB.

Translated from English version into Spanish by Sión Guitart, through

TRANSLATORS
WITHOUT BORDERS
